# Regional Variations in *Peucedanum japonicum* Antioxidants and Phytochemicals

**DOI:** 10.3390/plants13030377

**Published:** 2024-01-27

**Authors:** Neil Patrick Uy, Hoon Kim, Jajung Ku, Sanghyun Lee

**Affiliations:** 1 Department of Plant Science and Technology, Chung-Ang University, Anseong 17546, Republic of Korea; uyneilpatrick@gmail.com; 2 Department of Food and Nutrition, Chung-Ang University, Anseong 17546, Republic of Korea; hkim81@cau.ac.kr; 3 Forest Bioresources Department, National Institute of Forest Science, Suwon 16631, Republic of Korea; jajungku@korea.kr; 4 Natural Product Institute of Science and Technology, Anseong 17546, Republic of Korea

**Keywords:** antioxidant, *Peucedanum japonicum*, HPLC analysis, Phenolic compound

## Abstract

*Peucedanum japonicum* has long been a staple in East Asian cuisine. In the context of traditional medicine, various members of the *Peucedanum* genus have been investigated for potential medicinal properties. In laboratory settings, some compounds derived from this plant have shown antioxidant and anti-inflammatory properties—characteristics often associated with potential medicinal applications. This study aimed to determine which part of the *P. japonicum* plants cultivated on two Korean islands contains the most antioxidant compounds. This determination was made through assessments of total polyphenol content and total flavonoid content, coupled with evaluation of antioxidant activity via DPPH and ABTS assays. The results showed that the aerial parts contain a richer array of bioactive compounds and demonstrate superior antioxidant activity compared to their root counterparts in the plants from both islands. To characterize the phytochemicals underpinning this bioactivity, LC-MS/MS and HPLC analyses were carried out. These methods detected varying amounts of chlorogenic acid, peucedanol 7-*O*-glucoside, rutin, and peucedanol, with good separation and retention times. This study addresses the lack of research on the antioxidant activity of different parts of *P. japonicum*. The findings hold significance for traditional medicine, dietary supplements, and the development of functional foods. Understanding antioxidant distribution aids in the development of medicinal and nutritional applications, influences agricultural practices, and contributes to regional biodiversity-conservation efforts. The study’s geographical scope provides insights into how location impacts the concentration of bioactive compounds in plants. Overall, the results contribute valuable data for future research in plant biology, biochemistry, and related fields.

## 1. Introduction

Reactive oxygen species (ROS) are chemically reactive oxygen-containing molecules generated as byproducts of normal cellular metabolism and various environmental factors. An excess of ROS can induce oxidative stress, causing damage to cellular components such as proteins, lipids, and DNA [1]. This phenomenon has the potential to inflict damage on various molecules and cellular structures, thereby disrupting the proper functioning of organs and systems. The accumulation of ROS in the body results from both endogenous and exogenous mechanisms [2]. A growing body of evidence suggests the involvement of ROS in the physiopathology of numerous chronic diseases that require prolonged pharmacological treatment [1].

In the last few decades, an increasing number of individuals have been diagnosed with various chronic and acute diseases that are linked to oxidative stress [3]. Numerous studies have suggested that the consumption of an antioxidant-rich diet could reduce the impact of these diseases [4,5]. Plants inherently contain a wide array of antioxidant compounds as a natural defense against various stresses [6]. Consequently, incorporating these plants into our diet enhances our antioxidant intake, contributing, to an extent, to disease prevention [7].

In the study of plant-based antioxidants, coastal hog fennel, scientifically known as *Peucedanum japonicum*, has been a subject of interest in drug-discovery research due to its content of various bioactive compounds. This perennial herb from the Apiaceae family is extensively cultivated and utilized in East Asian countries for both food and medicinal purposes [8]. Although it is typically abundant in East Asian countries like Korea, Japan, and China, it can also be found in the Philippines and other Southeast Asian countries [9]. In Korean cuisine, this plant is referred to as “sikbangpung”, “bangpungnamul”, and “gaetgireumnamul” and is utilized as a “ssam” (wrap) or served as a pickled delicacy. Chinese traditional medicine has a long history of using this plant to address conditions such as fever, cough, and colds [10]. In terms of documented medicinal applications supported by scientific evidence, *P. japonicum* has exhibited effects against inflammatory diseases, rheumatoid arthritis, and neuralgia [11].

In the last twenty years, most studies have focused on the bioactive characteristics of *P. japonicum* roots. However, there has been a shift in the past ten years, with in-depth research increasingly focused on the aerial parts, rather than the roots [12,13]. Notably, no study has evaluated *P. japonicum*’s antioxidant activity in both its aerial and root sections, as far as the authors are aware. Furthermore, there is a paucity of studies that have directly contrasted the antioxidant activity of the chemical compounds in these two plant components. This plant has long been recognized for its rich content of phenolic acids, flavonoids, and other compounds known for their antioxidant properties. These compounds, abundant in various plant tissues, have been associated with a range of health advantages, including anti-inflammatory and anti-cancer properties [14,15]. The concentration or content of these compounds in a given plant varies considerably according to environmental conditions. Plants that grow in environmentally disturbed areas tend to accumulate more antioxidant compounds in their systems [16] Additionally, plants that are more exposed to sunlight tend to accumulate large amounts of antioxidants as a form of defense from UV rays [17]. The amount of antioxidant compounds also varies among different plant organs [18].

The present study examined compounds from two classes of phytochemicals that are prevalent in plants: polyphenols and terpenoids. Polyphenols, characterized by the presence of multiple phenolic (aromatic) rings, are known for their antioxidant properties [19]. Examples of polyphenols include chlorogenic acid and rutin. Chlorogenic acid, recognized for its presence in coffee, has been studied for its potential medicinal properties, including antioxidant [20,21], anti-inflammatory [22], neuroprotective [23], and weight-management effects [24]. Rutin has also demonstrated antioxidant [25,26], anticancer [27], and antiallergic [28] effects. With an established foundation in the exploration of polyphenols, as exemplified by chlorogenic acid and rutin, the focus now turns toward another class of plant-derived compounds—terpenoids. Peucedanol and its derivative, peucedanol 7-*O*-glucoside, are emerging as subjects of interest within this category. Terpenoids constitute a diverse group of organic compounds derived from the assembly of isoprene units [29]. They are found in various plants and are known for their structural diversity and biological activities. Terpenoids include compounds like monoterpenes, sesquiterpenes, diterpenes, and triterpenes. Peucedanol and peucedanol 7-*O*-glucoside share similar chemical structures, differing only in the presence of the glucoside ring attached to the C-7 position, as the name suggests. Likewise, peucedanol and peucedanol 7-*O*-glucoside have also been reported to possess antioxidant [30], antidiabetic [31], and antiplatelet-aggregation [32] activities. These compounds exhibit numerous bioactive properties, all stemming from their antioxidant activity and capacity to scavenge free radicals.

Antioxidant activity is usually assessed by both in vivo and in vitro approaches. However, in vitro assays are used more often in screening for antioxidant activity [33]. This preference may be attributed to the convenience of in vitro assays, which provide relatively quick results compared to their in vivo counterparts [34]. Hence, this study examined samples of *P. japonicum* grown in two different islands in Korea: four samples each of the aerial parts of *P. japonicum* from Ulleung Island (PAU), *P. japonicum* roots from Ulleung Island (PRU), aerial parts of *P. japonicum* from Jeju Island (PAJ), and *P. japonicum* roots from Jeju Island (PRJ). These samples were evaluated for their antioxidant activity using ABTS (2,2′-azino-bis (3-ethylbenzothiazoline-6-sulfonic acid) and DPPH (2,2-diphenyl-1-picrylhydrazyl). In addition, the phytochemical profile of the samples was assessed using liquid chromatography-tandem mass spectrometry (LC-MS/MS) and high-performance liquid chromatography (HPLC), using four standard compounds.

With the increasing interest in plant-based antioxidants, a significant research gap has emerged concerning variations in the phytochemical content and antioxidant activity of *P. japonicum* according to the region of origin. To address this gap, the present study aimed to quantify variations in antioxidant activity associated with environmental influences, as well as with the plant part tested. The results of this study can help to optimize the production of this plant and improve its market value both as a functional food and as a source of antioxidants.

## 2. Results and Discussion

### 2.1. Total Polyphenol and Total Flavonoid Content (TPC and TFC) Assays

When studying antioxidant activity in plants, researchers often analyze multiple plant parts, such as the aerial parts and the roots, to understand the distribution of antioxidants throughout the plant [35]. This approach is employed because different plant parts contain unique compounds and secondary metabolites. Hence, analyzing both aerial and root components allows for a more comprehensive assessment of the plant’s bioactivity. Antioxidant compounds, such as polyphenols, flavonoids, and phenolic acids, can vary in their distribution among plant tissues [36]. This study analyzed the antioxidant activity, TPC, and TFC of the methanolic (MeOH) extracts of four *P. japonicum* samples from plants growing on Jeju Island and Ulleung Island. Table 1 summarizes the results of the TPC and TFC assays. Notably, PAU exhibited the highest TPC, followed in descending order by PAJ, PRJ, and PRU. Similarly, PAU also had the highest TFC, followed in descending order by PRJ, PAJ, and PRU. The analysis of the results by one-way analysis of variance (ANOVA) revealed significant differences in the content of polyphenols among samples. However, no significant difference was observed between PAU and PRJ, although both samples were significantly different from PRU and PAJ in terms of TFC.

### 2.2. DPPH and ABTS Radical-Scavenging Assays

In antioxidant-activity assays, results are usually expressed as IC_50_ values, where lower IC_50_ values indicate higher activity in the extracts (Table 2). In the DPPH assay, PAJ exhibited the highest activity relative to the other samples, followed by PAU. The two remaining samples, PRU and PRJ, showed low activity, and IC_50_ values could not be generated for them. Conversely, all samples displayed activity in the ABTS assay. PAU had the lowest IC_50_ values, followed in order by PAJ, PRU, and PRJ. As expected, ascorbic acid, the positive control, showed the lowest IC_50_ values for both the DPPH and ABTS assays. The ANOVA results showed that the IC_50_ values of aerial-part samples, regardless of the region of origin, did not differ significantly from each other. Similarly, there was no observed significant difference in the IC_50_ values between the two root samples from both regions. However, both aerial part samples were statistically significant from both root samples. These findings suggest that the antioxidant activity of the plant is influenced more by the plant part used than by the region of origin. Moreover, the IC_50_ values of each sample were statistically different from the positive control in both assays.

The results of the TPC and TFC assays do not necessarily align with the results of the DPPH and ABTS assays. As previously noted, the predominant source of antioxidant activity in plant products is polyphenolic components, such as flavonoids, phenolic acids, tannins, and other phenolic compounds [37]. PAU, which had the highest content of polyphenols and flavonoids, also exhibited superior antioxidant activity in both the DPPH and ABTS assays. However, PRJ, despite having the second-highest polyphenol and flavonoid content, showed relatively low activity in both antioxidant assays. By contrast, the results from all assays conducted on PAJ and PRU coincide with each other. The nature of the samples is important here: PAU is the aerial part of *P. japonicum* grown on Ulleung Island, while PRU is its root counterpart; similarly, PAJ represents the aerial part of *P. japonicum* grown on Jeju Island, while PRJ is its root counterpart. The results of the present study contrast with those of a study that extracted compounds from the roots of the same plant using water and ethanol and tested the extracts for antioxidant activity [38]. Their results showed that the roots contain antioxidant compounds, as was evident in the DPPH and ABTS antioxidant assays. However, in general, plant roots tend to exhibit lower bioactivity compared to their aerial counterparts [39]. This difference is attributable to a higher concentration of antioxidant compounds in the aerial parts compared to the roots [40]. Aerial parts are primarily associated with photosynthesis, in which plants convert sunlight into energy and produce oxygen [41]. During photosynthesis, plants generate various secondary metabolites, including antioxidants, to protect themselves from oxidative stress caused by ROS produced in the process [42,43]. On the other hand, roots primarily serve functions related to nutrient absorption and anchoring the plant in the soil. While roots may still contain antioxidants, their concentration is generally lower compared to that of the aerial parts [44]. The antioxidant activity in the roots may be influenced by factors such as soil conditions, the presence of beneficial microbes, and exposure to various environmental stresses.

**Figure 1:**
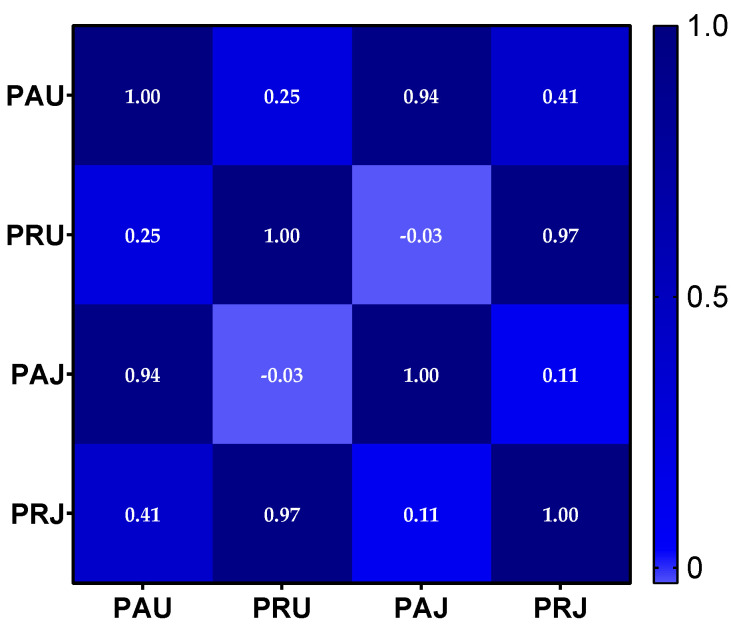
Heatmap of correlations between the samples analyzed.

To emphasize the similarities and differences between the different plant parts and to assess the effects of different harvest locations on the accumulation of bioactive compounds, Pearson correlation coefficients were generated from the values of the assays conducted (TPF, TFC, DPPH, and ABTS) and visualized in a heatmap (Figure 1). Briefly, each square in the plot represents the correlation between variables on each axis, with correlation values ranging from negative 1 to positive 1. Values closer to zero indicate no linear association between the variables. The closer the correlation is to 1, the more positively correlated the variables are: as one increases, so does the other, and the greater the value of the correlation coefficient, the stronger this relationship is. The heatmap shown below depicts a positive correlation for all samples analyzed, except for PAJ and PRU, which exhibit a negative correlation.

### 2.3. LC-MS/MS Analysis

**Figure 2:**
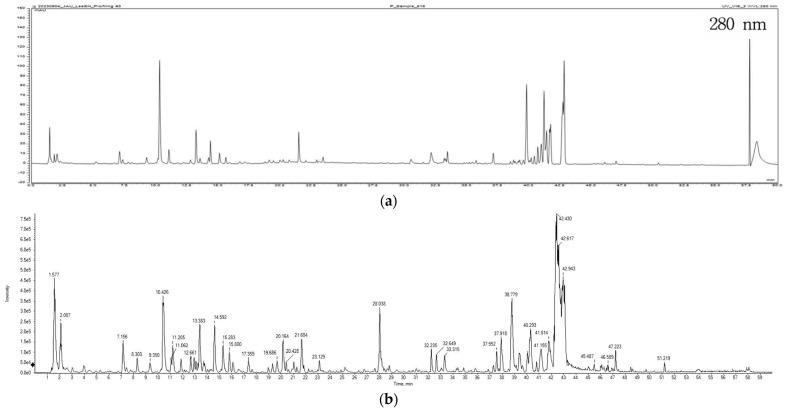
The UV chromatogram of PAJ at 280 nm (**a**) and the base peak chromatogram of PAJ analyzed in negative ion mode (**b**).

To identify the antioxidant compounds in the samples, LC-MS/MS analysis was conducted. The UV and base peak chromatogram of PAJ from the negative ionization mode analysis are depicted in Figure 2. The mass-to-charge ratio (*m*/*z*) was then identified using tandem MS. To identify the main peaks, a spectrum library and a web-based database were consulted. The negative ion mode analysis identified fifteen phytochemicals (Table 3). While the positive ion mode was also used, it failed to determine the same number of compounds. This discrepancy could have arisen because the compounds being analyzed are more suited for analysis in the negative ion mode. Compounds with specific functional groups, such as carboxylic acids, sulfonic acids, and phenols, tend to ionize well in negative ion mode. The results can also be affected by the polarity of the analytes [45]. Compounds that are inherently more polar or acidic may ionize better in negative mode. Conversely, basic or less polar compounds may exhibit better ionization in positive mode.

**Figure 3:**
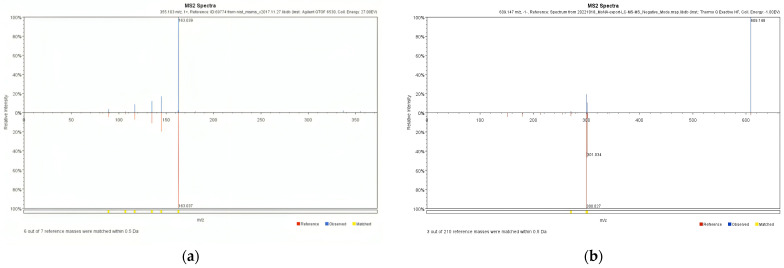
The MS/MS spectra of chlorogenic acid (**a**) and rutin (**b**).

The MS/MS spectra of the aforementioned compounds are presented in Figure 3. These spectra depict the distribution of ions based on their *m*/*z* values. The configuration of peaks in the mass spectrum revealed the distinct fragmentation or ionization patterns characteristic of the analyzed compound [46,47]. Coupled with the retention time obtained from the LC, these results made it possible to identify the compounds. Through this procedure, the analysis detected two of the four compounds of interest (chlorogenic acid and rutin). This result coincides with the results of a previous study that also detected abundant chlorogenic acid (**1**) and rutin (**3**) in the seeds of *P. japonicum* by LC-MS/MS [48].

### 2.4. HPLC Analysis

**Figure 4:**
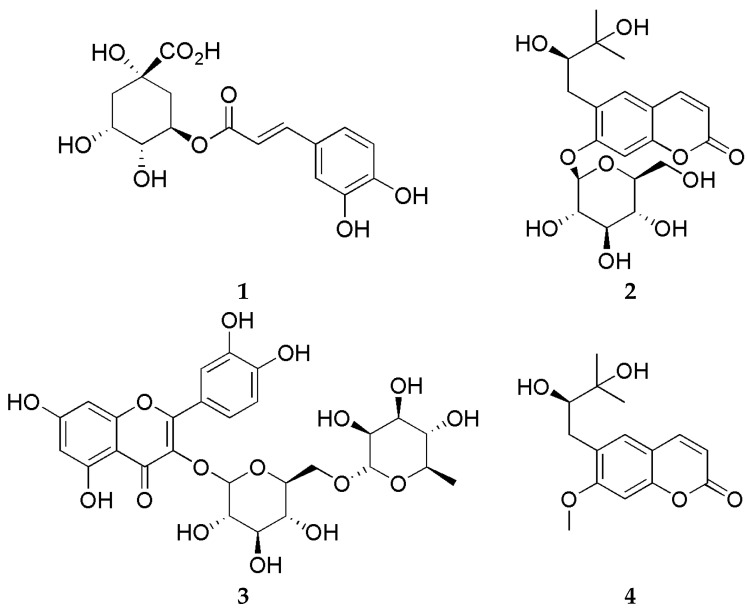
The chemical structures of chlorogenic acid (**1**), peucedanol 7-*O*-glucoside (**2**), rutin (**3**), and peucedanol (**4**).

**Figure 5:**
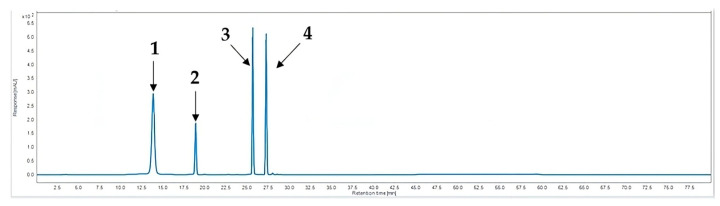
HPLC chromatogram of chlorogenic acid (**1**), peucedanol 7-*O*-glucoside (**2**), rutin (**3**), and peucedanol (**4**).

The HPLC analysis employed four standard compounds, namely chlorogenic acid (**1**), peucedanol 7-*O*-glucoside (**2**), rutin (**3**), and peucedanol (**4**). The chemical structures of the standard compounds are depicted in Figure 4. The standard compounds were well separated and demonstrated favorable retention times (Table 4) (Figure 5).

**Figure 6:**
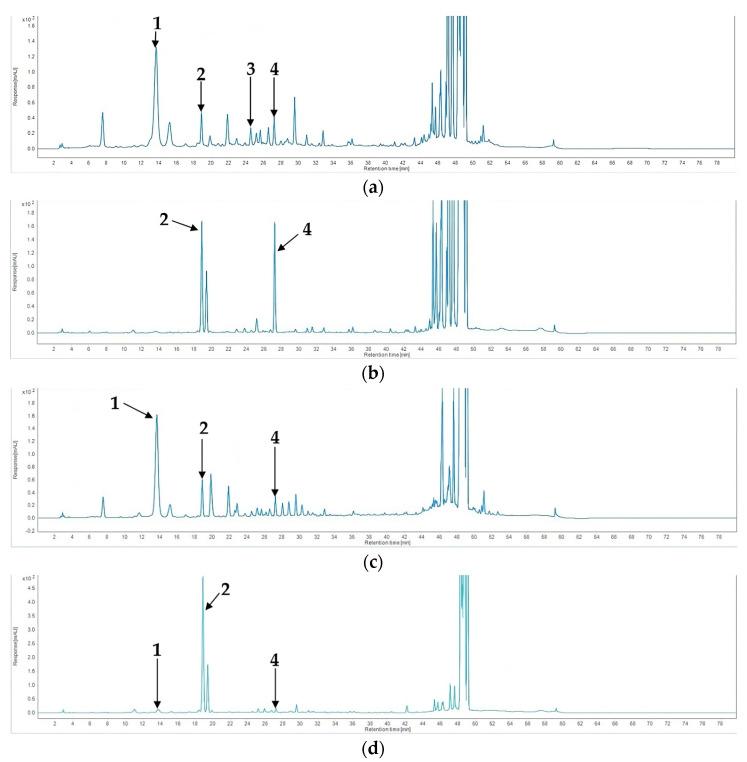
HPLC chromatograms of PAU (**a**), PRU (**b**), PAJ (**c**), and PRJ (**d**) extracts (**1**, chlorogenic acid; **2**, peucedanol 7-*O*-glucoside; **3**, rutin; and **4**, peucedanol).

The HPLC analysis results aligned with the LC-MS/MS results, wherein chlorogenic acid (**1**) and rutin (**3**) were both detected. PAU was the only sample in which all standard compounds were detected (Figure 6). Conversely, PRU was found to contain peucedanol 7-*O*-glucoside (**2**) and peucedanol (**4**), while chlorogenic acid (**1**) was detected only in trace amounts. The remaining two samples, PAJ and PRJ, were both found to contain chlorogenic acid (**1**), peucedanol 7-*O*-glucoside (**2**), and peucedanol (**4**) only. Overall, samples from Jeju Island were found to contain the highest concentrations of the compounds, while the samples from Ulleung Island contained lower concentrations (Table 5). The statistical analysis revealed significant differences in the content of each compound, with a confidence level of 95%. One-way ANOVA revealed that the plant part and the region of origin significantly affect the content of every compound except for peucedanol 7-*O*-glucoside (**2**), for which no significant difference was observed among the samples originating from Ulleung Island (PAU and PRU). 

A previous study conducted phytochemical profiling on *P. japonicum* leaves and successfully identified and isolated fifteen compounds [30]. They detected all standard compounds assessed in this study, but chlorogenic acid (**1**) and rutin (**3**) were found to be the major antioxidant constituents. Another study also detected chlorogenic acid (**1**) and its derivatives in the leaves of *P. japonicum*, which could explain the bioactive properties of those leaves [49]. Compared to the results of the present study, the samples of aerial parts analyzed in that study contained relatively higher amounts of chlorogenic acid (**1**), while rutin (**3**) was detected only in PAU. The detection of these compounds is consistent with the results of the present study, but the amounts detected vary; such differences are to be expected, as the samples used were from different sources.

The present study aimed to bridge a critical knowledge gap concerning the plant components of *P. japonicum*, specifically whether the aerial parts or the roots exhibit the highest antioxidant activity. The results have implications for traditional medicine, dietary supplements, and the development of functional foods. Antioxidants are often associated with health benefits, and understanding their distribution in plant parts holds significant potential for usefulness in medicinal and nutritional applications [50,51]. From an agricultural standpoint, understanding antioxidant distributions may have implications for cultivation practices. This knowledge could influence decisions related to plant breeding, crop management, or the selection of plant varieties with enhanced antioxidant content [52]. Furthermore, given the diverse geographical origins of the samples, any observed variations in antioxidant activity offer invaluable insights into the influence of geographical factors on the concentrations of bioactive compounds in plants. Moreover, the findings are valuable for conservation efforts and understanding regional biodiversity. Lastly, the results add to the body of scientific knowledge by providing data on antioxidant potential and phytochemical profiling in specific plant parts. This information can serve as a reference for future research in plant biology, biochemistry, and related fields.

## 3. Materials and Methods

### 3.1. Plant Materials and Extracts

**Figure 7:**
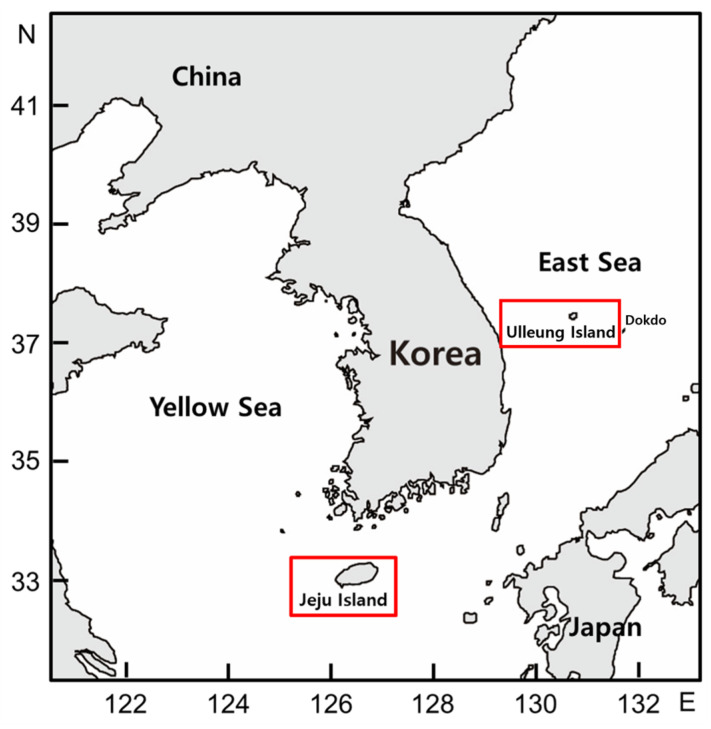
Collection sites of coastal hog fennel.

The MeOH extracts from coastal hog fennel (*P. japonicum*) were obtained from the Korea Research Institute of Bioscience and Biotechnology (KRIBB), Daejeon, Korea. These samples are referred to as PAU (*P. japonicum* aerial parts from Ulleung Island), PRU (*P. japonicum* root from Ulleung Island), PAJ (*P. japonicum* aerial parts from Jeju Island), and PRJ (*P. japonicum* root from Jeju Island). Jeju Island and Ulleung Island are two of the most famous and largest islands in Korea (Figure 7).

### 3.2. Chemicals and Apparatus

HPLC was performed using an Agilent Technology 1260 Infinity II (Santa Clara, CA, USA) and INNO C18 column (250 × 4.6 mm, 5 μm) equipped with a pump, an auto-sampler, and an Agilent Variable Wavelength (VW) Detector (Santa Clara, CA, USA). The HPLC-grade solvents water (H_2_O), trifluoroacetic acid (TFA), and acetonitrile (ACN) were purchased from J. T. Baker (Radnor, PA, USA). Chlorogenic acid (**1**), peucedanol 7-*O*-glucoside (**2**), rutin (**3**), and peucedanol (**4**) were provided by the Natural Product Institute of Science and Technology (www.nist.re.kr; accessed on 10 December 2023), Anseong, Republic of Korea.

### 3.3. Preparation of Samples and Standard Solutions for HPLC Analysis

To achieve a concentration of 30,000 ppm, 30 mg of each of the *P. japonicum* extracts was dissolved in 1 mL of HPLC-grade MeOH. For the standard compounds, 1 mg each of chlorogenic acid (**1**), peucedanol 7-*O*-glucoside (**2**), rutin (**3**), and peucedanol (**4**) was dissolved in 1 mL of HPLC-grade MeOH to achieve a concentration of 1000 ppm. The solutions were subjected to sonication for 10–15 min and filtered using a polyvinylidene fluoride (PVDF) membrane filter with a pore size of 0.45 μm. Subsequently, the standard solutions were serially diluted to obtain concentrations suitable for calculations in the quantitative analysis.

### 3.4. TPC Assay

The determination of TPC in the four *P. japonicum* extracts followed a previously established method, with slight modifications [53]. Initially, a stock solution of each sample with a predetermined concentration was serially diluted to yield three to five solutions with different concentrations. Afterward, 60 μL of each sample was added to a 96-well microplate. Next, 40 μL of Folin–Ciocalteu reagent (Sigma-Aldrich, St. Louis, MO, USA) were added to each of the wells containing samples. Subsequently, 100 μL of 7.5% Sodium carbonate (Na_2_CO_3_) was added and the samples were allowed to react at room temperature in the dark for 30 min. Finally, the absorbance of the samples was measured at 760 nm using a microplate reader (Epoch; BioTek, Winooski, VT, USA). TPC was calculated utilizing a standard curve constructed with varying concentrations of tannic acid.

### 3.5. TFC Assay

The TFC of the samples was assessed using a modified version of a method described in a previous study [54]. Initially, a stock solution of each sample with a determined concentration was serially diluted to yield three to five solutions with different concentrations. Afterward, 100 μL of each sample was added to a 96-well microplate. Thereafter, 100 μL of 2% aluminum chloride hexahydrate (AlCl_3_·6H_2_O) was added to each of the wells containing samples. The microplate plate was then incubated for 10 min at room temperature. Following this step, the absorbance of the samples was measured at 430 nm using a microplate reader (Epoch; BioTek, Winooski, VT, USA). The TFC was then determined using a standard curve constructed with varying concentrations of quercetin.

### 3.6. DPPH Radical-Scavenging Assay

The DPPH radical-scavenging assay was based on a previously described method [55]. The assay began with the preparation of a working solution containing 0.2 mM DPPH, which was produced by diluting the initial DPPH stock solution with 95% MeOH. Before the start of the main assay, a stock solution of each sample with a predetermined concentration was serially diluted to yield three to five solutions with different concentrations. Subsequently, 10 µL of the test solution was added to the wells of a 96-well microplate. Afterward, 200 µL of the DPPH working solution was added to the same wells. This process was repeated three times for accuracy. Following thorough mixing on a microplate shaker (Mx4, FINEPCR, Gunpo, Republic of Korea), the solutions were allowed to incubate in darkness for 30 min. Following this step, the absorbance was measured at wavelengths of 514 nm. Ascorbic acid was used as the standard. To calculate the DPPH free-radical-scavenging activity of the samples (%), the equation below was used:

DPPH radical-scavenging activity (%) = (Blank O.D − Sample O.D)/Blank O.D × 100

### 3.7. ABTS Radical-Scavenging Assay

The ABTS radical-scavenging assay was based on a previously described method [56]. The ABTS stock solution was created by dissolving 7.4 mM ABTS and 2.6 mM potassium persulfate in deionized water. To prepare the working solution, the stock solution was diluted in water until it reached an absorbance of 1.0 at 730 nm. Before the main assay began, a stock solution of each sample with a predetermined concentration was serially diluted to yield three to five solutions with different concentrations. Subsequently, 10 µL of the test solution was dispensed into the wells of a 96-well microplate. Afterward, 200 µL of the ABTS working solution was added to the same wells. The mixtures underwent 30 s of shaking on a microplate mixer (Mx4, FINEPCR, Gunpo, Republic of Korea). The microplates were then incubated in the dark for 30 min at room temperature. Finally, the absorbance at 734 nm was measured using a microplate reader (Epoch Microplate Spectrophotometer, BioTek, Winooski, VT, USA). To calculate the ABTS free-radical-scavenging activity of the samples (%), the equation below was used:

ABTS radical-scavenging activity (%) = (Blank O.D − Sample O.D)/Blank O.D × 100

### 3.8. LC-MS/MS Conditions

The analysis was conducted using an LC system composed specifically of a Thermo Vanquish UHPLC machine equipped with a Waters Cortex T3 column (150 mm × 2.1 mm, particle size 1.6 μm). The temperature was maintained at 45 °C. The mobile phase consisted of water (eluent A: 0.1% HCOOH) and acetonitrile (eluent B: 0.1% HCOOH), with a gradient applied and a flow rate set at 0.25 mL/min. The mass spectrometric analysis was performed on a Triple TOF 5600^+^ System (AB SCIEX, USA) featuring a heated electrospray ion source (H-ESI). The mass spectrometer operated in positive and negative ion modes, generating survey full-scan MS spectra (*m*/*z* 100–1500) using a quadrupole system with a resolution setting of 70,000. The spray voltage was set at 3.5 kV for the positive ion mode. The top fifteen most intense precursor ions underwent MS2 fragmentation with spectra acquisition at a resolution setting of 17,500. Additional MS parameters included a capillary temperature of 320 °C, sheath gas at 50 AU, sweep gas at AU, and auxiliary gas at 10 AU.

### 3.9. HPLC Conditions

The quantitative analysis of the four *P. japonicum* samples was carried out by slightly modifying a previously reported method [57]. A YMC Pack-Pro C18 column (250 mm × 4.6 mm, 5 μm) maintained at a column temperature of 30 ℃ was equipped in the HPLC system. The method utilized a gradient elution with a mobile phase comprised of 0.1% TFA in water (A) and ACN (B). The gradient elution conditions were as follows: 90% A from 0 min to 10 min, 70% A at 30 min, 50% A at 40 min, and 0% A from 45 min to 55 min. The mobile phase flow rate was set to 1 mL/min, with an injection volume of 10 μL. Lastly, the detector wavelength was set at 330 nm.

### 3.10. Calibration Curves

The chlorogenic acid (**1**), peucedanol 7-*O*-glucoside (**2**), rutin (**3**), and peucedanol (**4**) standard solutions underwent serial dilution, with a minimum of five concentrations used to establish their respective calibration curves. The linearity of each calibration curve was determined based on the correlation coefficient (*r*^2^). The content of the target compound was calculated using the equation derived from the calibration curve. In the calibration correction function for the five compounds, the *x*-axis (µg/mL) represented the concentration and the *y*-axis represented the peak area; the mean value (n = 3) ± standard deviation was utilized for substitution.

### 3.11. Statistical Analysis

The results were expressed as the mean ± SD. To ensure the reliability, statistical power, and robustness of the experimental findings, each experiment was conducted three times (in triplicate). The data were tested for normality and log normality before being treated with the statistical tests described above. The data was analyzed using one-way ANOVA followed by Tukey’s post hoc test. GraphPad Prism 8.0.2 statistical software (GraphPad Software, Boston, MA, USA) was used to perform all statistical tests and generate the corresponding graphs. Values with *p* < 0.05 were considered to be statistically significant. 

## 4. Conclusions

According to the assay results, the aerial parts sourced from Ulleung Island exhibited the highest efficacy, as well as the highest polyphenol and flavonoid content. In the HPLC analysis, four phytochemical compounds were used as standards to quantify the compounds present in the samples. The results of this analysis align cohesively with those of the LC-MS/MS analysis. In summary, this study aimed to explore the complexities of the interactions between bioactivity, phytochemical profiles, and geographic factors. Following an examination of both the aerial and root sections, the results showed an overall hierarchy of polyphenol and flavonoid content, with the highest amounts found in PAU. Antioxidant assays also demonstrated the general superiority of PAU in terms of antioxidant content, while the surprising discordance in the results for PRJ emphasizes how complex antioxidant interactions are. A vast chemical tapestry is revealed by this phytochemical profiling, with PAU containing the most varied chemicals. This study has implications for conservation efforts, nutraceutical agriculture, and health applications. This work provides a basis for further investigations into the complex ecology of *P. japonicum*, particularly in the areas of plant biology and biochemistry.

## Figures and Tables

**Table 1:** Comparison of the TPC and TFC in PAU, PRU, PAJ, and PRJ.

Sample	TPC (mg TAE/g Extract)	TFC (mg QE/g Extract)
PAU	18.89 ± 6.2 ^a^	6.88 ± 4.9 ^a^
PRU	8.29 ± 3.8 ^d^	4.64 ± 1.2 ^c^
PAJ	15.58 ± 4.3 ^b^	5.83 ± 2.5 ^b^
PRJ	10.34 ± 5.4 ^c^	6.37 ± 4.6 ^a^

Note: TAE, tannic acid equivalent; QE, quercetin equivalent. The results are expressed as the mean ± standard deviation (SD) (n = 3). Mean ± SD values in each column followed by different letters indicate values that are significantly different from each other (*p* < 0.05).

**Table 2:** Radical-scavenging assessment of antioxidant activity in PAU, PRU, PAJ, and PRJ using DPPH and ABTS assays.

Sample	Concentration (mg/mL)	DPPH		ABTS	
		Scavenging Activity (%)	IC_50_ (mg/mL)	Scavenging Activity (%)	IC_50_ (mg/mL)
PAU	10	68.72 ± 0.11	5.59 ± 0.13 ^a^	83.90 ± 0.26	2.71 ± 0.08 ^a^
	5	46.94 ± 0.78		66.03 ± 0.66	
	2.5	36.03 ± 2.93		48.85 ± 0.75	
	1.25	18.91 ± 1.25		30.50 ± 2.55	
	0.625	7.70 ± 0.76		18.71 ± 2.36	
PRU	10	13.73 ± 0.23	-	50.97 ± 0.20	9.57 ± 0.14 ^b^
	5	7.11 ± 1.61		38.96 ± 1.41	
	2.5	5.02 ± 0.17		31.88 ± 1.04	
	1.25	4.61 ± 0.80		25.05 ± 0.88	
	0.625	2.86 ± 0.96		21.54 ± 0.61	
PAJ	10	58.07± 1.23	8.92 ± 0.07 ^a^	83.41 ± 0.33	3.15 ± 0.35 a
	5	24.27 ± 2.45		67.31 ± 3.49	
	2.5	13.44 ± 2.68		43.44 ± 3.75	
	1.25	9.34 ± 5.05		29.00 ± 1.83	
	0.625	4.65 ± 2.46		24.11 ± 0.95	
PRJ	10	18.76 ± 3.35	-	50.86 ± 0.52	9.60 ± 0.26 ^b^
	5	12.39 ± 0.79		38.48 ± 1.09	
	2.5	7.96 ± 3.70		30.22 ± 0.12	
	1.25	7.18 ± 5.04		26.16 ± 0.86	
	0.625	3.50 ± 4.31		22.76 ± 0.36	
*^a^*AA	0.2	68.83 ± 0.84	0.09 ± 0.00 ^b^	86.12 ± 1.46	0.10 ± 0.00 ^c^
	0.16	49.62 ± 3.95		63.67 ± 0.52	
	0.12	38.01 ± 0.84		49.41 ± 0.18	
	0.08	19.17 ± 0.34		27.96 ± 0.31	
	0.04	3.35 ± 0.93		15.47 ± 0.10	

Note: *^a^*AA, Ascorbic acid. The results are expressed as the mean ± SD (n = 3). Mean ± SD values in each column followed by different letters indicate that the values are significantly different from each other (*p* < 0.05).

**Table 3:** Proposed structures based on LC-MS/MS analysis (negative mode).

Retention Time (min)	Molecular Weight	Tentative Identification
7.16	354.1	Neochlorogenic acid
7.44	138.0	Protocatechuic aldehyde
9.34	338.1	3-*p*-Coumaroylquinic acid
10.42	354.1	Chlorogenic acid
11.19	354.1	Cryptochlorogenic acid
12.93	354.1	1-Caffeoylquinic acid
13.38	338.1	Coumaroylquinic acid
14.39	164.0	*p*-Coumaric acid
15.28	368.1	3-*O*-Feruloylquinic acid
15.80	338.1	3-*p*-Coumaroylquinic acid
19.29	464.1	Isoquercetin
19.31	610.2	Rutin
19.63	464.1	Hyperoside
20.43	434.1	Guaiaverin
21.67	610.2	Hesperidin

**Table 4:** The calibration-curve equations for chlorogenic acid (**1**), peucedanol 7-*O*-glucoside (**2**), rutin (**3**), and peucedanol (**4**).

Compound	t_R_	Calibration Equation ^a^	Correlation Factor, *r*^2 b^
**1**	13.80	Y = 11.617x *−* 34.047	0.9989
**2**	18.83	Y = 26.264x + 14.37	0.9999
**3**	25.62	Y = 18.475x + 5.1917	1.0000
**4**	27.20	Y = 13.764x + 27.381	0.9999

^a^ Y = peak area, X = concentration of standards (mg/mL). ^b^ *r*^2^ = correlation coefficient of five calibration data points (n = 3). t_R_ = retention time.

**Table 5:** Quantification of chlorogenic acid (**1**), peucedanol 7-*O*-glucoside (**2**), rutin (**3**), and peucedanol (**4)** in the four *P. japonicum* samples.

Sample	Content (mg/g ext.)				
	1	2	3	4	Total
PAU	5.03 ± 0.14 ^a^	1.31 ± 0.07 ^a^	0.79 ± 0.04	0.26 ± 0.01 ^b^	6.96 ± 0.23
PRU	tr	4.89 ± 0.00 ^b^	ND	1.76 ± 0.02 ^d^	6.65 ± 0.02
PAJ	5.83 ± 0.12 ^b^	1.69 ± 0.05 ^a^	ND	0.29 ± 0.00 ^c^	7.81 ± 0.18
PRJ	0.34 ± 0.05 ^c^	14.33 ± 0.33 ^c^	ND	0.13 ± 0.00 ^a^	14.8 ± 0.38

tr: trace; ND; not detected. The results are expressed are the mean ± SD (n = 3). Mean ± SD values in each column followed by different letters indicate that the values are significantly different from each other (*p* < 0.05).

## Data Availability

The data presented in this study are available on request from the corresponding author.
